# Towards a dynamic, comprehensive conceptualization of dyslexia

**DOI:** 10.1007/s11881-023-00297-1

**Published:** 2024-01-13

**Authors:** Maryanne Wolf, Rebecca J. M. Gotlieb, Sohyun An Kim, Veronica Pedroza, Laura V. Rhinehart, Maria Luisa Gorno Tempini, Sue Sears

**Affiliations:** 1 Department of Education, University of California, Moore Hall 2123, 405 Hilgard Avenue, Los Angeles, CA 90095-1521 USA; 2 School of Medicine, University of California, San Francisco, USA; 3 Michael D. Eisner College of Education, California State University, Northridge, USA

**Keywords:** Dyslexia, Heterogeneity, History of dyslexia, Reading brain circuit, Reading subtypes, Risk and resiliency

## Abstract

Here we build from the central strength of the existing definition of dyslexia—its emphasis on neurobiological origins—and proffer a set of seven core principles for a new, more comprehensive conceptualization of dyslexia. These principles derive from two major research directions: (1) the still evolving history of attempts to explain dyslexia, including in varied writing systems; and (2) the study of the reading brain circuit, its development, and its genetic and environmental influences. What emerges from connecting these two directions is a dynamic conceptualization of dyslexia that incorporates the extensive research on the heterogeneity of dyslexia and the interdependent contributions of multiple biological and socio-cultural risk and preventive factors. A new definition of dyslexia, therefore, needs to transcend both past unitary characterizations and past assumptions based largely on the English orthography. Such a conceptualization references the ways that different languages interact with the reading brain circuit to produce different sources of reading failure. Similarly, the characteristics and consequences of dyslexia that have been considered as secondary sequela (e.g., reduced reading comprehension, social-emotional issues) should be part of a more comprehensive narrative. Of critical importance, any definition of dyslexia should clarify persisting misconceptions that associate dyslexia with a lack of intelligence, potential to learn, or talents. Thus, the overall purpose of such a definition should serve as an instrument of knowledge and an enduring reason for pursuing growth in reading for the individual, the educator, and the public.

Not unlike Emily Dickinson’s famous line, “Tell all the truth, but tell it slant; Success in Circuit lies” (Dickinson, 1890, p. 506), in this paper we will approach the need for a new definition of dyslexia circuitously through two directions that intersect in a “circuit” Dickinson never anticipated. First, we will examine the insight-rich history of attempts to understand dyslexia over the last century; and second, we will connect this history to our current, evolving understanding of the reading brain circuit. Together, these different slants of knowledge provide the basis for a more comprehensive, dynamic conceptualization of dyslexia that reflects its inherent, brain-based heterogeneity and the complex impact of external, socio-emotional, and demographic variables on reading failure and reading achievement.

## A history of dyslexia: “tell all the truth, but tell it slant”

Anyone who hopes to understand dyslexia through the history of its research may be flummoxed at the outset. There are few more convoluted historical records than that for dyslexia with its multiple names, definitions, and differing explanations of its underlying cognitive mechanisms. Yet, each of these names and explanations offers an insight into a given epoch’s understanding of reading and reading challenges. Just as Alberto Manguel (2014) chose to call his memorable book *A History of Reading* to emphasize that such a history can be told in many ways, we wish to follow his example here for the same reasons. In this way we dismiss no past research perspective for its narrower lens at that time, nor do we attempt to be inclusive in such a brief space. Rather, not unlike the story of the blind men and the elephant, we hope to show how a selected, cumulative history of the varied hypotheses unexpectedly contributes to a more comprehensive understanding of what connotes dyslexia in different individuals.

### Visual and perceptual hypotheses

The fact that few phenomena have been given more differing names and more causal explanations over the last century than dyslexia has good, important reasons that will emerge later. The first use of the term “*dyslexia*” (derived from the Latin morpheme *dys* for ‘difficult’ and the Greek root *lexis* for ‘word’) by German ophthalmologist Berlin was chosen to denote difficulties with words by various adult patients. Later in the nineteenth century, two physicians, Pringle Morgan (1896) and Hinshelwood, used another term, *word-blindness* (derived from “Wort-Blindheit” by German ophthalmologist, Kussmaul, 1878) to describe an unexpected failure in children to develop reading based on an inability to see words correctly. Thus, the first causal explanations begin in the visual system, an explanation that will come in and out of this history and resurface in a more nuanced way most recently by Yeatman (e.g., White et al., 2019).

Neurologist Orton (1928) conducted some of the most influential dyslexia research during the early twentieth century and promptly replaced the term word blindness with the term *strephosymbolia*. Like others to come, Orton wished to depict what he considered to be a different underlying locus of reading failure: i.e., lateralization confusions between the brain’s two hemispheres that caused difficulties in processing symbols. In a similar vein, various neurologists and neuroscientists over time emphasized the contributions of other brain regions to indicate the role that areas like the limbic system, cerebellum (Stoodley & Schmahmann, 2009), and thalamus (Llinas, 1993) can play in visual representation, memory, and the precise timing of component parts (Wolf, 1991; Wolff, 1993) needed during reading. Again, new names like *amnesia visualis verbalis* for key memory issues and *dyschronia* for timing issues (Llinas, 1993) reflected the hypotheses of the researcher about what was most disruptive to the acquisition of reading in dyslexia.

Although disruptions in the visual system continued to be the predominant explanations in the first two-thirds of the twentieth century (Vellutino, 1979), other sensory systems were implicated in *auditory processing disorders*, as discussed early on by Lucy Fildes (1921) and evolved over time in several directions. For example, by mid-century, *multi-modality* (Blank & Bridger, 1964) explanations focused more attention on the connections between and among the visual and auditory systems, as well as to cognitive processes that governed these connections—like the ability to hold information in working memory and sequence verbal information. This research was the precursor for later important work on the role of executive functions in reading (e.g., Cirino et al., 2019; Kim, 2023; Swanson & Kong, 2018). During the 1970s, one of the most heatedly debated hypotheses, the *temporal processing disorder*, was rooted in speech and language pathology research and focused on the temporal, acoustic dimensions of auditory perception of the phoneme (Tallal & Piercy, 1973).

### Phonological-based hypotheses

The most critical opponents of the latter view emerged from the field of psycholinguistics and would change the history of dyslexia. The explosion of work on the role of phonological processes in reading and in dyslexia emerged from early studies by Bond and Dykstra (1967) and from a program of research by psycholinguists working in Bell Laboratories and later Haskins Laboratories (e.g., Kavanaugh & Mattingly, 1972; Liberman et al., 1989; Liberman & Shankweiler, 1979; Malins et al., 2016). The hypothesis was that a failure within language systems, specifically phonological processes involved in the representation of phonemes and the developing meta-awareness of them, was the major linguistic source of disruption in dyslexia. This view, the *phonological-core deficit hypothesis*, eclipsed previous emphases on *perceptual deficit hypotheses* that involved visual and/or auditory systems (Vellutino, 1979) and became a dominant perspective till the present.

The investigations into the relationship between the phonological components of language and reading yielded significant advances in our understanding of the important role of phonemic awareness (PA) in learning the alphabetic principle in reading, as well as its utility in the early identification of reading difficulties and the teaching of beginning reading (Bradley & Bryant, 1983; Ramus, 2003; Shaywitz & Shaywitz, 2020; Torgesen et al., 1999; Washington & Seidenberg, 2021). The importance of phoneme-level representation in beginning reading is intrinsically connected to the alphabetic nature of the English writing system. More specifically, the ability to map the individual distinctive sounds of spoken English (i.e., phonemes) to the symbols (i.e., graphemes) representing these sounds is critical in the development of decoding and spelling. Phonemic awareness was considered such a crucial skill that it was identified by the National Reading Panel Report (NICHD, 2000) as one of five essential elements of effective reading instruction and is included in the goals of most interventions in dyslexia. Stretching across five decades to the present, there have been more scientific papers on this hypothesis than for any other unitary hypothesis into the causes of dyslexia (e.g., Torgesen, 2018; Wagner et al., 2019).

### Neurological hypotheses

Although few today dispute the importance of phonological processes in reading and reading failure, a parallel direction of research from neurology and the cognitive neurosciences emphasized the presence of other areas of cognitive weakness causing or contributing to dyslexia symptoms. Perhaps the most influential of these hypotheses originated with the seminal research by behavioral neurologist Norman Geschwind on the “Disconnexion Syndrome in Animals and Man” (Geschwind, 1965). Before cognitive sciences became their own area of study, Geschwind used basic models of the brain to depict what caused *acquired* reading failure or *alexia* that resulted from discrete areas of stroke or brain injury. He used these models to convey the then novel hypothesis that different *disconnections* among the varied reading-related areas (e.g., vision and language regions within and between the two hemispheres) could underlie different forms of reading failure, as evident in *alexia with* and *without agraphia*.

Tucked within this overarching view of adult alexia is one of the less known aspects of developmental dyslexia's history. With these models as context, Geschwind hypothesized that *color naming*, which requires the connection of the visual and language systems in order to retrieve a name for abstract symbols like color, would be a powerful predictor of dyslexia in children. Color naming, he speculated, would expose the inability to connect the key reading systems to retrieve a name and thus predict dyslexia. Although by itself this hypothesis proved incorrect, his student, pediatric neurologist Denckla, and neuropsychologist Rudel went on to find that the *speed* of connecting these systems in naming various symbols did, in fact, predict dyslexia (Denckla & Rudel, 1976).

This was the beginning of two related but diverging directions of research spawned by the prolific work of Geschwind and his students, particularly Denckla and Galaburda.

Denckla pursued more cognitive-behavioral differences that could explain the predictive capacity of naming speed—that is, the rapidity of retrieval for visually presented symbols—for individuals with dyslexia. Investigating what Denckla and Rudel termed *Rapid Automatized Naming* (RAN), Denckla and her student Wolf (Wolf, 1991; Wolf & Denckla, 2005), along with Bowers (Wolf & Bowers, 1999; Wolf & Bowers, 2000a), and multiple other researchers (see reviews in Norton & Wolf, 2012; McWeeny et al., 2022) found extensive evidence for a “second deficit” in RAN or naming speed in individuals with dyslexia. Despite efforts to explain this as a phonological-based weakness, Wolf and Bowers (Wolf & Bowers, 1999; Wolf & Bowers, 2000a) demonstrated that there are children with RAN issues without PA involvement, as well as children with PA differences only. Most importantly, the most severely impaired children possessed both RAN and PA issues, among other challenges. Similar studies proliferated: e.g., using taxonomic classification methods, O'Brien et al. (2012) found analogous subtypes in a sample of 600 children. Extensive cross-linguistic research from various writing systems replicated the presence of an independent RAN weakness that was more predictive than PA in more transparent languages (Di Filippo et al., 2005; Ibrahim, 2015; Katzir et al., 2004; Share, 2008; Spencer & Hanley, 2003; Torppa et al., 2012; Traficante, 2023) and in other orthographies like Chinese (Tan et al., 2005).

There is an important, insufficiently understood historical caveat. Wolf and Bowers (Wolf & Bowers, 1999; Wolf & Bowers, 2000b) first used the term “Double-Deficit Hypothesis” to include, but go beyond, unitary phoneme-based hypotheses. However, from the outset they conceptualized the three most common subtypes as placeholders for research into the various possible sources of disruption *underlying* RAN, as well as into multiple other vulnerabilities beyond RAN and phonology. The RAN and later RAS (Rapid Alternating Stimulus) tasks (Wolf & Denckla, 2005) were conceptualized then and now as assessing the speed with which various visual and language processes are connected, any one or more of which can disrupt the rapidity of retrieval (Wolf & Obregón, 1992). In this view, therefore, naming speed involves a mini-circuit that includes many of the same cognitive and linguistic processes in the larger reading circuit. This is the underlying basis of its predictive capacities. A RAN deficit is thus an *index* of various possible sources of disruption, not the actual disruptor.

### Neurobiological causes of dyslexia

Work on the neurobiological and structural anatomical changes underlying dyslexia remains insufficient, but has important insights to build from, particularly the research program by Geschwind’s student, Al Galaburda. He and his colleagues pursued post-mortem, brain tissue studies as a fundamental way to understand dyslexia and other neurodevelopmental disorders (e.g., Galaburda et al., 1985). They (Galaburda, 1989) performed the first of such studies thirty years ago and showed that *cytoarchitectonic* changes caused by cellular migration and formation in Perisylvian regions (see also Stein, 2001) characterized the limited number of autopsied brains of individuals with dyslexia. Subsequently, using both brain and animal genetic models, Galaburda and his colleagues contributed greatly to an understanding of dyslexia at the cytoarchitectonic level by showing how disruptions in early cell migration in particular areas can impede later processing of written language.

These pioneering studies, although limited in sample size, provide evidence for the hypothesis that cortical development anomalies in key language-related brain regions could underlie Geschwind’s “disconnections”. These same anomalies may also underlie the lack of activation and connectivity in the neural networks associated with dyslexia on functional imaging studies (Norton & Wolf, 2012; Pugh et al., 2000).

Early and ongoing genetic studies buttress this hypothesis: for example, those genes most consistently associated with dyslexia and language processing, such as KIAA0319, are genes that regulate neuronal migration (Paracchini et al., 2006). Recent clinical and neuropathological studies in older patients with atypical neurodegenerative disease and selective language and reading symptoms complement this evidence. Miller and colleagues reported that patients with a form of aphasia characterized by a phonological variant show a higher rate of developmental dyslexia than the general population (Miller et al., 2019). The brain tissues of three of these older individuals with developmental dyslexia were recently analyzed post-mortem at the UCSF Brain bank and showed distinct cortical anomalies in well-studied language regions, in addition to the expected pathology of Alzheimer patients (Miller et al., 2019).

Taken together, these studies support a highly relevant hypothesis that early-life neurodevelopmental changes might influence not only educational attainment in childhood, but also life-long brain development. To be sure, we need more studies that investigate this longitudinal view, alongside the root causes of dyslexia at the histological level and the molecular biology level (see Krishnamurthy et al., 2019; Pugh et al., 2014). That said, the notion that neurodevelopmental, cortical changes can occur throughout the most complex and vulnerable areas in frontal, temporal, and parietal regions could both reconcile many of dyslexia’s history of different hypotheses described above and also help explain the heterogeneity in the subtyping research described below.

### Cognitive and neuroimaging subtype research

Early neuropsychological work in the 1970s on subtypes of individuals with learning disabilities indicated the presence of discretely different characteristics not neatly explained by single causes (see Fletcher & Satz, 1985; Satz & Morris, 1981). The early subtype classifications became increasingly methodologically sophisticated through the work of Morris, Fletcher, Lyon, Francis, and their colleagues (e.g., Morris et al., 1998). Morris et al. (1998) completed one of the most important studies of subtypes of students with reading disability. They found nine subtypes based on students’ scores on standardized measures of PA, working memory, RAN, IQ, developmental history, and teacher reported behavior at one point in time. Like the findings by Wolf and Bowers (1999), the most impaired of their subtypes had multiple challenges, including both PA and RAN. Recent influential research by Ozernov-Palchik et al. (2017) in a group of over one thousand kindergarteners showed subgroup stability over two years. The four at-risk groups included students with weaknesses in PA, in RAN, in multiple areas including PA and RAN, and in below-average performance across all skills. There remains insufficient research on how individual subgroups change over time, particularly with appropriate intervention.

An additional, recent contribution to the research on subtypes involves brain imaging. For example, research teams headed by Gabrieli and Hoeft (Norton et al., 2014), Gaab and Ozernov-Palchik (Ozernov-Palchik et al., 2016) and Lyytinen (Lyytinen et al., 2005; Torppa et al., 2012) used brain functional imaging techniques to investigate discrete differences for different subtypes in underlying brain regions, particularly groups characterized by PA and RAN weaknesses. A different focus in a recent study using sophisticated psychophysics and neuroimaging techniques showed distinct profiles of basic auditory (non-speech sounds) and speech sound perception challenges in children with dyslexia. These profiles were associated with focal changes in specific regions of the superior temporal cortex (Qi et al., 2023). If these results were confirmed, they could reconcile the auditory and phonological processing hypotheses as different anatomical variants of the same condition. All of these studies provide critical links among neurobiological, neuroanatomical and clinical subtyping efforts (e.g., Norton et al., 2014).

Within the context of this heterogeneity have come several other new directions that involve a new look at an old hypothesis: the role of the visual system. Reminiscent of dyslexia’s “word-blindness” origins, recent work by Yeatman and his colleagues has documented the critical role of the brain’s visual word form area and visual spatial skills, such as spatial attention and the rapid encoding of visual information, in reading development (Kubota et al., 2019; Ramamurthy et al., 2021; Ramamurthy et al., 2022; White et al., 2019). The visual word form area (alternatively referred to as the occipital-temporal junction) has been at the fore of research on reading development by cognitive neuroscientists, including Dehaene and Cohen (Dehaene et al., 2004; McCandliss et al., 2003). Addressing one of the more persistent questions in dyslexia’s history, Rueckl et al. (2015) have shown in imaging studies that this brain region is implicated in dyslexia in multiple languages.

### Dyslexia in languages other than English

The cumulative importance of these latter directions has direct implications for any future definition of dyslexia that goes beyond unidimensional views and indeed beyond hypotheses based solely on the English written language system. As discussed in detail early on by Aro and Wimmer (2003), by Ziegler and Goswami (2005), and more recently by Daniels and Share (2018), most studies on dyslexia, particularly those that concentrate on the phonological-deficit hypothesis, have been conducted in English. Although the ability to map the individual phonemes of spoken English to the graphemes representing these sounds is critical in the development of decoding and spelling in alphabetic writing systems, not all alphabetic writing systems are the same, and further not all writing systems are alphabetic. It is important for research in dyslexia to account for differences in the phoneme-grapheme relationships not only in shallow orthographies like Spanish, German, Dutch, Italian, and Finnish that are more transparent and consistent than deep orthographies like English and French, but also in different writing systems like Chinese and Japanese Kanji (Bolger et al., 2005). It is important, therefore, to acknowledge the crucial role played by cross-linguistic variations in orthographic transparency in both children’s reading development and in our understanding of dyslexia’s risk, manifestations, and progression (Kim & Petscher, 2013; Seymour et al., 2003). As one example, the important role of phonological weaknesses in the less transparent English language is not the same for children reading in more transparent languages (Anthony & Francis, 2005; Landerl et al., 2013).

By contrast, the important role of automaticity or the speed of connection between visual and language systems in reading, as indexed by RAN, appears to be more prevalent both in transparent orthographies (Georgiou et al., 2016; López-Escribano et al., 2018; Traficante, 2023) and in non-alphabetic writing systems like Chinese. Indeed, in investigations with Chinese subjects, Tan, Eden, Perfetti and others (Tan et al., 2005) likened RAN to a universal predictor because all writing systems require rapid connections between visual and language regions of the brain. In other words, RAN requires some of the same processes and same speed and seriation requirements that develop in the early reading brain’s circuit regardless of writing system. Exemplifying this in a logosyllabary, Tan et al. (2005) found that phonological awareness made a relatively weaker contribution to Chinese children’s reading development, than naming speed, writing, and orthographic awareness.

Although many subtyping efforts are based on processes underlying reading like PA and RAN, other efforts classify subtypes based on different aspects of reading performance. For example, Lovett (Lovett, 1987; Lovett et al., 1988) was one of the first researchers to classify readers on the basis of rate and/or accuracy in reading performance. More recently, Capin et al. (2021) identified three subtypes based on differences in word reading and listening comprehension deficits: moderate deficits in both word reading and listening comprehension, severe deficits in word reading but moderate deficits in listening comprehension, and severe deficits in listening comprehension but moderate deficits in word reading. Similarly, Kim and colleagues (2021) classified Korean readers into subgroups using the same analytical methods as in the Capin et al. (2021) study, but with a classification based on the severity of the deficit areas. Compton (2021) attributed such differences in Kim et al.’s (2021) criteria to the cross-linguistic difference in orthographic transparency, since Korean is more transparent in its orthography than English. The essential point that we wish to incorporate in any conceptualization of dyslexia is that both the known predictive factors and external variables, particularly language and orthography variation (Pugh & Verhoeven, 2018), affect our ability to predict reading development and its different forms of disruption.

### Multifactorial hypotheses

In the last decade, a growing body of research on multifactorial models has been examining the effect of known predictive factors in the presence of different external factors. In multifactorial models, dyslexia, and especially its severity, is viewed as resulting from a combination of and/or interaction among risk factors, including phonological processing, RAN, and oral language problems, all of which, like dyslexia, are dimensional in nature and manifest in varying degrees of severity. Very importantly as noted by Catts and Petscher (2022), these complex interactions are also influenced by environmental factors like quality of instruction, language variation, parental support, and the individuals’ background. Thus, multi-factorial models tend to represent dyslexia as probabilistic rather than deterministic: i.e., risk factors in conjunction with protective factors increase or decrease the likelihood of dyslexia and its severity. Risk or symptomology of learning disabilities, which can include dyslexia, is increased by factors like co-morbid developmental language, attentional, or anxiety disorders (Nelson & Harwood, 2011), exposure to trauma (Duplechain et al., 2008), consciousness of reading disability stigma (Daley & Rappolt-Schlichtmann, 2018), and teachers misunderstanding of dialectical differences (Washington & Seidenberg, 2021). Conversely, there are benefits of high-quality mentoring (Haft et al., 2019), interventions that increase feelings of motivation (Lovett et al., 2021), emotional resilience (Goldberg et al., 2003; Zheng et al., 2014), and growth mindsets (Andersen & Nielsen, 2016), familial identity and practices concerning reading (Willingham, 2015), and exposure to dynamic conversations at home (Romeo et al., 2018). These factors can decrease dyslexia symptomology, are associated with the development of parts of the reading brain circuit, and improve students’ feelings about reading, in some case even controlling for effects of socioeconomic status. Within that context, in efforts to understand and define dyslexia, alongside known weaknesses, we must consider possible assets associated with dyslexia (e.g., Palser et al., 2021; Sturm et al., 2021) and individual strengths and protective factors. In other words, we must consider the role of changing environmental factors and social-emotional assets in the dynamic manifestations of dyslexia.

One of the very important additional variables that has emerged in multifactorial approaches concerns the genetic history of the individual and how this may contribute to children’s reading profiles. Early on, Pennington and Smith (1983) reviewed all the genetic evidence about dyslexia available at the time and documented that dyslexia is strongly heritable, associated with multiple genes, and also influenced by environment. More recently, Powers, Gaab and their colleagues discovered that home literacy environments differentially influence children’s reading profiles, based on their familial history of dyslexia (Powers et al., 2016).

At present there are several large-scale, genome-wide association studies that investigate more precise relationships between particular genes and specific deficits associated with dyslexia. For example, Eising et al. (2022) studied genetic relationships to word-reading, and Dębska, Pugh and their colleagues examined the influence of familial history of dyslexia in children’s neural correlates of phonological awareness (Dębska et al., 2016). Black, Hoeft, and their colleagues identified maternal history of dyslexia to be a risk factor for developmental dyslexia (Black et al., 2012). An important addition to the existing genetic studies involves the subject populations. For example, ongoing studies by members of the Gruen lab are investigating the genetic relationships of deficits like RAN in less studied populations of African-American and Hispanic-American children and other groups underrepresented in reading research (Gialluisi et al., 2019; Truong et al., 2019).

Nevertheless, as Grigorenko (2022) points out in her recent review, although genetic history is the “single dominant force shaping individual differences in reading acquisition” (p.104), more robust evidence from different countries, languages, and writing system are warranted in order to generalize the evidence on the role of these genetic factors across various cultures and populations. Within that context, recent research programs by Hoeft and by Gorno-Tempini are investigating the effects of language variation, particularly children’s early bilingual experiences, as well as genetic history, on our understanding of the important contributors to reading development and its potential disruptors. The latter include parental education and family financial circumstances as important contributors to students’ reading development and dyslexia risk.

Along similar lines, some multifactorial models emphasize not only the importance of different language backgrounds and socioeconomic circumstances, but also the different pathways in children’s reading development that are due to multiple cognitive skills involved in reading comprehension. For example, Kim (2017) used the Direct and Indirect Effect Model of Reading model to reveal that children’s working memory, theory of mind, inference, comprehension monitoring, vocabulary, grammar, word reading, and listening comprehension all collectively contributed to reading comprehension in multiple ways. Such findings are important to any conceptualization of dyslexia because they expand our understanding of reading development and failure by including language variation, the underlying roles of cognitive processes, and their combined effects on various aspects of reading, including comprehension.

Still other multifactorial studies include factors that remain insufficiently incorporated in our understanding of dyslexia, particularly protective factors like resilience and social-emotional sequelae. Risk-resilience models of dyslexia include both causal factors that put children at risk for dyslexia and resilience factors that influence more positive reading outcomes. In these models, protective factors, such as an individual’s cognitive and socio-emotional strengths and resiliency (Haft et al., 2016; Palser et al., 2021; Sturm et al., 2021), environmental factors such as high-quality early reading instruction (Fletcher et al., 2019), and the confluence of these protective factors (Gotlieb et al., 2022) mitigate against poor reading in at-risk populations. Risk-resilience models that consider protective factors in combination with risk factors move our conceptualization of dyslexia away from a more exclusive focus on deficits, towards the need for understanding the role of an individual’s strengths in reading outcomes.

### A summary of a history of dyslexia

Examining the history of almost any conceptualization is a humbling exercise, but one with lessons that deepen our knowledge and help to guide our next steps. The history of our efforts to understand dyslexia, like the science of reading, is evolving not only with our research, but also with an understanding that each accretion of knowledge has real-life implications for the individuals with dyslexia, their teachers, families, and communities. In the next section, we examine another evolving branch of knowledge: specifically, the study of the reading brain circuit, its development, and its influences. To forecast, we believe that the coterminous examination of the history of dyslexia and the science underpinning the reading brain’s development will propel our field’s efforts to provide a more comprehensive understanding of the heterogeneous nature of dyslexia and indeed of the complexity of reading that underlies it.

## A brief examination of the reading brain circuit: “success in circuit lies”

The current definition of dyslexia by the International Dyslexia Association begins by stating that “dyslexia is a specific learning disability that is neurobiological in origin.” Although much of the research cited in this paper underscores the need for revising various other aspects of the definition, particularly the emphasis on only one typical form of deficit, the neurobiological basis remains the single most important core of the definition. We will return to the other parts of the definition in our conclusions.

It is our view that an understanding of the neurobiological basis of dyslexia is best situated within an understanding of what comprises the reading brain circuit and the not-so-simple insight that our brain was never meant to read (Wolf, 2007, 2018). Our ability to learn this evolutionarily new cognitive capacity required that the brain learn to build a new circuit by connecting multiple distributed regions across both hemispheres, all the lobes and layers of the brain, including the cerebellum (our evolutionarily older “little brain), and various key subcortical regions (Dehaene, 2009). The sum of these connected networks is a reading circuitry that has the ability to add ever more sophisticated cognitive, linguistic, and affective processes over time. Indeed, in its expert state, the original reading brain is a circuit of circuits. Further, the brain is not the only organ that supports reading; the definition states that dyslexia is *neurobiological* in origin, rather than neurological, in part because of the recruitment of the eyes, ears, and even viscera in reading.

We believe that the very complexity of the reading brain circuit offers another lens on why reading development is such an achievement and why it can go awry in many different ways, often as not before children even learn to read (Langer et al., 2017). A brief examination of several of the major precursor regions will help to underscore some of the ways that reading can be difficult for different children for different reasons from genetic to environmental. Indeed from the outset, multiple genes have been associated with dyslexia with some estimates of as much as 70% heritability of dyslexia (Erbeli et al., 2021). Similarly, as seen in the last section’s recent history, a range of environmental factors from family socioeconomic status, language backgrounds, to quality of literacy in the home have been shown to contribute to the experience of learning to read, to the severity of dyslexia, and to the development of the reading brain. Indeed, the major components that will make up the circuit—like visual, cognitive, linguistic, and affective processes—begin their development in the first years of life when both genetic factors and the environment are highly interactive. In reading as in life, nature and nurture influence both the precursors of reading and the various ways it can be disrupted (Gotlieb et al., 2022).

To begin with language, processes undergirding phonology and semantic knowledge to syntax and morphology are essential pre-reading skills that grow exponentially, *depending* on the language environment of the child and the family’s genetic history. Underpinning these linguistic processes are the temporal, parietal and frontal lobes of the brain with different areas specialized for different types of phonological, semantic, morphological, and grammatical knowledge. Oral language, with its involvement of speech and motor regions, requires a range of capacities that allow young children to represent phonemes in ways that differ from the way other non-speech sounds are processed. These fine-tuned phonological processes recruit multiple regions within the left frontal, temporal, and parietal lobes, such as the supramarginal gyrus, as well as right hemisphere areas responsive to aspects of oral language like rhyme and intonation (Hickok & Poeppel, 2007).

The contributions of visual areas, like the previously discussed visual word form area, underpin various orthographic skills that prepare the child to recognize and eventually represent the visual symbols and visual patterns of the child’s particular writing system (Borghesani et al., 2021). Adjacent to the visual regions is the angular gyrus, an area that Geschwind (1965) believed key to the coordination of reading-related areas and whose disruption he associated with alexia with agraphia. The angular gyrus is involved in the connection and translation of visual information processed in the occipital region to semantically comprehensible input in the temporal regions (Pugh et al., 2000).

The cognitive components that contribute to the reading circuit include myriad areas involved in executive function processes like working memory to the formation and consolidation of background knowledge in long-term memory within various areas, particularly the medial temporal gyrus. The effortful control of attention and the ability to direct and focus it is another pre-reading skill that is associated with a frontoparietal network of cortical, subcortical and mid-layer regions in the brain.

Still another set of precursor skills that require more emphasis in the early reading circuit are the contributions of emotional engagement and the forerunners of introspection, both of which help the developing pre-reader make important affective associations to reading, as well as learn to think carefully about stories and ideas heard from books read to them. As discussed by Wolf (2007, 2018), these associations provide not only the ideal foundation for reading development, but also a protective factor that can affect the severity of dyslexia. The insula, buried deep within the cortex, is a critical part of the reading brain that is associated with making these strong emotional associations with reading, and, in conjunction with prefrontal structures, supports the growth of reflection and the child’s own thinking over time.

Although the development of each and all of these precursor processes is evidence enough of the complexity inherent in reading development, the circuit cannot function properly without processes responsible for the rapid deployment and temporal coordination of these precursor regions with each other. Only when each component can work together at almost automatic speeds will fluent decoding and fluent comprehension become possible. The thalamus, a subcortical area of the brain, and the cerebellum are both critical for the precise timing and temporal integration of all the components of the reading circuit, just as the angular gyrus and other connective regions are involved in their coordination.

**Fig. 1:**
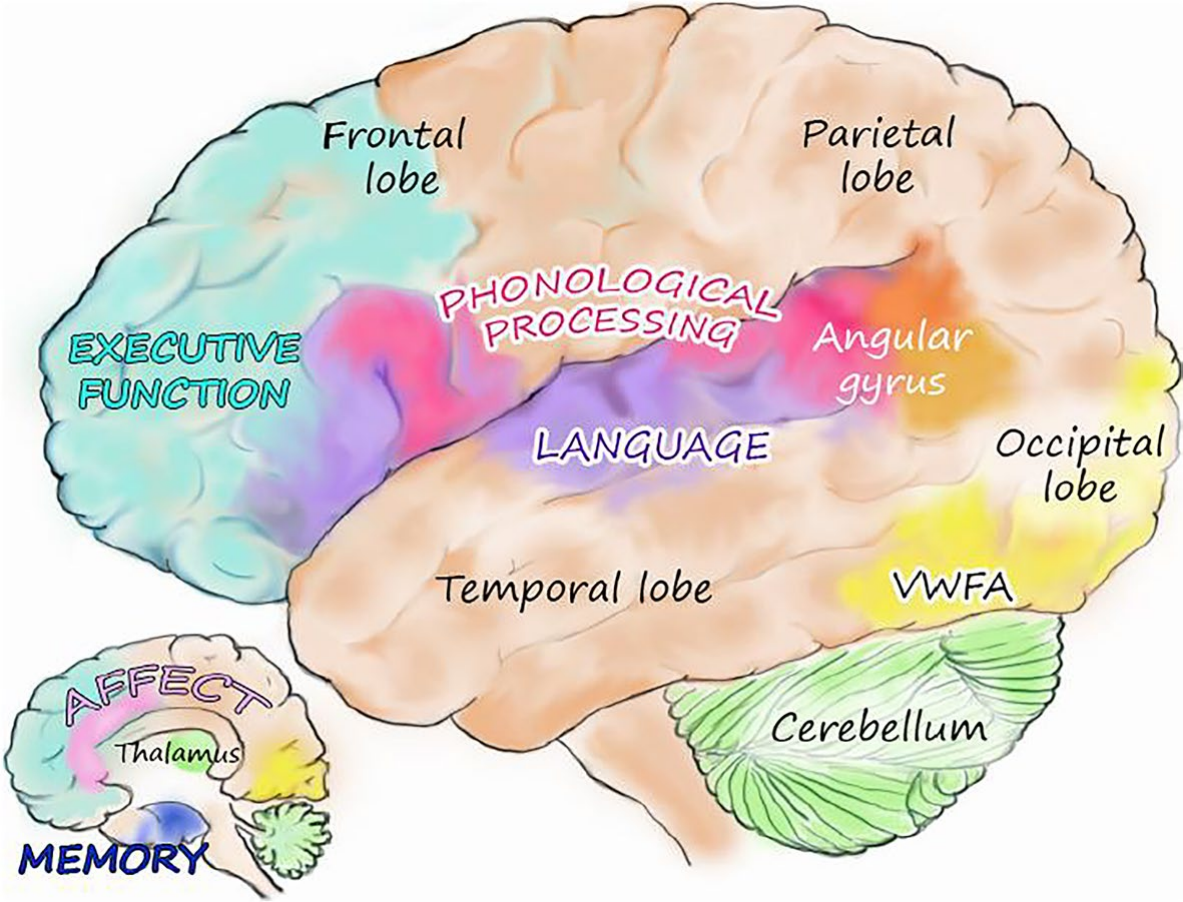
Regions in the reading brain circuit

With the help of neuroscientist, artist, and cerebellum expert Catherine Stoodley, Fig. 1 provides a schematic representation of the major regions underlying processes involved in the reading brain circuit.

The overarching leitmotiv of this brief overview and of Fig. 1’s depiction is that written language is a stunning example of the brain’s ability to make all manner of new connections that were never genetically programmed like oral language and vision. The reading circuit represents an extraordinary leap forward in the brain’s use of its neuroplasticity to make new circuits for novel human inventions like literacy and numeracy. The complexity and the plasticity of the new circuit, however, contribute to both its Achilles-like strength and vulnerability. For, with such complexity and neuroplasticity-based adaptational ability, no one source of disruption and no one environmental influence will be sufficient to capture all forms of its failure in all individuals. Just as the history of dyslexia is pockmarked by various explanations, the circuit is subject to different causes of disruption or failure and different influences.

## Bringing history and the reading brain circuit together

**Fig. 2:**
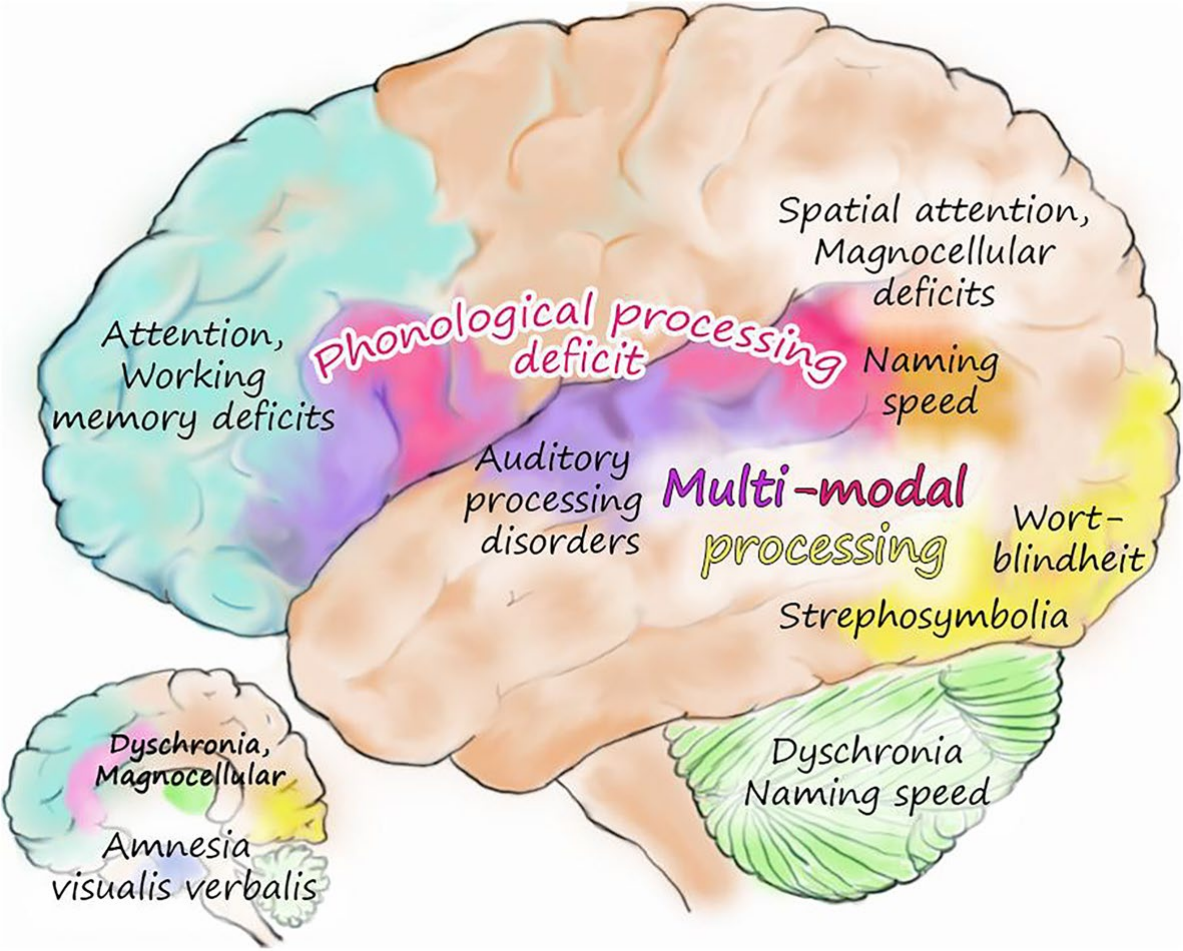
A Map of the history of explanations for dyslexia

Returning to our initial use of Emily Dickinson’s “circuitous” approach to truth, we wish now to connect the two sections of this paper. In Fig. 2, we have graphed many of the names used in the history of dyslexia onto the reading brain circuit. Note that when the various names for dyslexia over the last century and a half are mapped on the underlying regions, they provide a surprisingly close approximation of the reading brain circuit. In other words, if something can go wrong in the reading brain circuit, at some point in the history of dyslexia, it has gone awry and become the basis of the next new name and next causal hypothesis. The two sections of this paper converge to provide the basis for the heterogenous nature of dyslexia, the first of several principles that go beyond the original definition of dyslexia.

By looking at both history and the reading brain circuitry together, there are several principles that we believe are key for a new conceptualization and eventual definition of dyslexia. First, there will be different manifestations of dyslexia depending on what is most vulnerable in the circuit for a given language. Although the natural desire in science for parsimony has made the more unitary hypotheses of dyslexia dominate its history, the cumulative history of dyslexia and the reading brain itself reveal that there will be no single form of dyslexia, an understanding further confirmed by cross-linguistic and cross-writing system studies. There will be different profiles of characteristics among children with dyslexia across languages with weaknesses in phoneme awareness, the processes underlying RAN, and potentially other language, visual, and executive functions presenting in different combinations, depending on the writing system (Daniels & Share, 2018; Share, 2021).

Second, the reality that dyslexia is developmental and influenced by both genetic and environmental factors adds further complexity. The influences of language variation and language/literacy background, cultural heritage, type and intensity of instruction and intervention will impact how dyslexia manifests in a given individual over time. Although dyslexia can be effectively remediated, especially when the particular areas of weakness are identified early on in individual learners (Lovett et al., 2017; Ozernov-Palchik & Gaab, 2016), the neurobiological differences in the brain’s organization remain largely the same across the lifespan. It is in this sense that dyslexia is not so much a reading disorder, but rather a different organization of the brain. This organization disadvantages the acquisition of the human species’ relatively new invention of reading through various disruptions to the reading circuit, including neurodevelopmental anomalies before the circuit is formed, as well as can give advantages in other arenas when enabled to do so.

Third, and an outgrowth of the second principle, the differences in brain organization that have historically been conceptualized largely in terms of weaknesses and deficits should be researched and described in conjunction with concomitant strengths and explicitly disassociated with any lack of intelligence. Indeed, the areas of weakness will be impacted by the opportunities individuals with dyslexia have to develop their potential strengths, particularly in visual-spatial skills (Schneps et al., 2012; Von Karolyi et al., 2003; Wolff & Lundberg, 2002), interpersonal skills (Palser et al., 2021; Sturm et al., 2021), and declarative memory (Hedenius et al., 2013). Currently, research into educational practices to support the strengths associated with dyslexia is still limited, especially for marginalized youth (Blanchett, 2010). Although there is some evidence to suggest that dyslexia is not associated with greater creativity (Erbeli et al., 2022), the history of individuals with dyslexia illustrates the over-representation of people with dyslexia in the arts, entrepreneurship, and other professions that rely on abilities to think and perceive “outside the box” (Logan, 2009). Therefore, asset-based teaching pedagogies for students with dyslexia (Orkin et al., 2022) must be further explored in future research.

It is critical to the societal understanding of dyslexia that, although it is typically described as a specific learning disability, dyslexia is a learning difference that becomes a disability to the extent that environments are disabling. There are social and emotional sequela often associated with environments that make students feel ashamed or lacking in intelligence. Noted long ago by Orton and by Geschwind (1982) in a paper memorably titled “Why Orton was Right”, there is a greater vulnerability to mental health challenges such as anxiety and depression (Maughan & Carroll, 2006; Sturm et al., 2021) among individuals with dyslexia. It is essential, therefore, both for the public and for the individuals with dyslexia that the content of our definition helps to combat negative stereotypes about dyslexia and appreciate the role of environment, including educational and socioeconomic context, in contributing to dyslexia.

Such a definition should ensure that teachers, parents, and students know that individuals with dyslexia are lacking neither in intelligence nor potential talents; and that with appropriate, evidence-based, structured literacy interventions, particularly those with emphases on resilience, persistence, and esteem (Gotlieb et al., 2022; Haft et al., 2016; Hendren et al., 2018; Lovett et al., 2017, 2022; Lyon & Goldberg, 2023; Morris et al., 2012; Rappolt-Schlichtmann et al., 2018), they can and will learn to read, even when early environments did not provide optimal supports. The history of rigorous reading intervention studies in the last twenty years underscores how well we can remediate children at risk for dyslexia, regardless of their race, socioeconomic background, or IQ. There are no silver bullets to intervention, but individuals with dyslexia can and will learn to read, particularly when provided interventions that are early, evidence-based, structured, multicomponential, and explicitly taught (Lovett et al., 2017; Lovett et al., 2022; Lyon & Goldberg, 2023; Morris et al., 2012). In the process, such interventions increase the social-emotional wellbeing of struggling readers (Traficante et al., 2017).

## Conclusions: Towards a dynamic, comprehensive conceptualization of dyslexia

The history of our understanding of dyslexia has continuously been evolving, just as is our research into the reading brain circuit’s complex origins and its potentially even more complex future in the digital culture. What is constant is *change*, not only in our concepts of dyslexia, but also within how individuals with dyslexia change over time according to how they are taught, how they are perceived, and how their individual strengths are enabled by society. Thus, the very definition of dyslexia itself must be dynamic and designed to change with newer knowledge and ever more sophisticated and comprehensive insights into its diagnosis and instruction. Here, we have discussed the existence of different subtypes of dyslexia that may depend on an individuals’ unique neurodevelopmental profile, language system, social-emotional and environmental factors, strengths, and preventative factors. Although the prevalence of particular subtypes of dyslexia may vary over time and context, the heterogeneity of dyslexia will persist.

Our contributions to efforts to construct a new definition will be in the form of principles we hope will be embedded by those ultimately responsible for writing this definition.

1. Dyslexia represents a unique organization of the human brain that is characterized by both disadvantages and advantages, both of which are influenced by neurodevelopmental, genetic, and environmental factors that have no relationship to intelligence.
2. The most common disadvantages include unexpected difficulties in the acquisition of accurate and/or fluent reading, spelling, and comprehension.
3. The most common predictive factors include independent or combined weaknesses in phonological components of language and processes indexed by naming speed, either of which can co-occur with multiple other factors such as executive function and visual-orthographic-related processes.
4. There will be different manifestations of dyslexia depending on what is most vulnerable in the reading circuit for a given language and writing system.
5. Both the risk for and developmental course of dyslexia are influenced by social, emotional, and environmental factors that range from socioeconomic background and language variation to emotional vulnerabilities and educational experience.
6. Although typically classified as a specific learning disability, dyslexia should never be associated with a lack of intelligence, talent, or effort. With appropriate, evidence-based, structured literacy instruction that is provided as early as possible, individuals with dyslexia can learn to read and develop their full potential.
7. Dyslexia can change over time, particularly when the strengths and advantages of its unique differences in brain organization are fostered alongside preventive factors that emphasize resiliency, persistence of effort, and respect for the potential of every individual.
